# Correlates of Calcaneal Quantitative Ultrasound Parameters in Patients with Diabetes: The Study on the Assessment of Determinants of Muscle and Bone Strength Abnormalities in Diabetes

**DOI:** 10.1155/2017/4749619

**Published:** 2017-09-19

**Authors:** Francesco Conti, Stefano Balducci, Luca Pugliese, Valeria D'Errico, Martina Vitale, Elena Alessi, Gerardo Salerno, Carla Iacobini, Stefano Menini, Lucilla Bollanti, Antonio Nicolucci, Giuseppe Pugliese

**Affiliations:** ^1^Department of Clinical and Molecular Medicine, “La Sapienza” University, Via di Grottarossa 1035, 00189 Rome, Italy; ^2^Diabetes Unit, Sant'Andrea Hospital, Via di Grottarossa 1035, 00189 Rome, Italy; ^3^Metabolic Fitness Association, Via Nomentana 27, Monterotondo, 00015 Rome, Italy; ^4^Radiology Unit, Sant'Andrea Hospital, Via di Grottarossa 1035, 00189 Rome, Italy; ^5^Laboratory of Clinical Chemistry, Sant'Andrea Hospital, Via di Grottarossa 1035, 00189 Rome, Italy; ^6^Center for Outcomes Research and Clinical Epidemiology (CORESEARCH), Via Tiziano Vecellio 2, 65124 Pescara, Italy

## Abstract

**Objective:**

Quantitative ultrasound (QUS) provides an estimate of bone mineral density (BMD) and also evaluates bone quality, which has been related to increased fracture risk in people with diabetes. This study aimed at assessing the correlates of calcaneal QUS parameters in diabetic subjects encompassing various degrees of micro and macrovascular complications and a wide-range of peripheral nerve function.

**Methods:**

Four hundred consecutive diabetic patients were examined by QUS to obtain values of broadband ultrasound attenuation (BUA), the speed of sound (SOS), quantitative ultrasound index (QUI), and BMD.

**Results:**

Among surrogate measures of complications, sensory and motor nerve amplitude and heart rate response to cough test and standing correlated with QUS parameters at univariate analysis, together with age, body mass index (BMI), waist circumference, lipid profile, and renal function. Multivariate analysis revealed that BUA, SOS, QUI, and BMD were independently associated with age, male gender, hemoglobin A_1c_, BMI (or fat, but not fat-free mass), and somatic and autonomic nerve function parameters.

**Conclusions:**

These data indicate that peripheral nerve dysfunction is associated with worse QUS parameters, possibly contributing to increased fracture risk in diabetes. The positive relation of QUS measures with adiposity needs further investigation. This trial is registered with ClinicalTrials.gov (NCT01600924).

## 1. Introduction

It is now widely recognized that diabetes mellitus (DM) is associated with an increased risk of fractures, which seems to be dependent on mechanisms at least partly differing from those associated with senile or postmenopausal osteoporosis [1]. A meta-analysis of 16 studies showed a relative risk for hip fractures of 6.94 in type 1 DM (T1DM) and 1.38 in type 2 DM (T2DM) compared to subjects without DM [2]. Interestingly, bone mineral density (BMD), the strongest predictor of fractures in subjects with osteoporosis [3], was found to be reduced in patients with T1DM in most, but not all studies, whereas cross-sectional surveys in subjects with T2DM reported normal or even increased BMD, despite the overall increase in fracture risk [2]. Moreover, increased fracture risk in T2DM patients remained after adjustment for BMD [4–6] and also for falls [4, 6, 7], which are more frequent in older individuals with T2DM than in their nondiabetic counterparts [8]. While the lower fracture risk in T2DM versus T1DM may be explained by the normal-to-increased bone mass, the apparent discrepancy between preserved BMD and increased fracture risk in individuals with T2DM and the higher fracture risk than that estimated from BMD variation in subjects with T1DM have prompted the hypothesis that DM patients have reduced bone quality [1, 2].

Bone quality is determined by (a) bone architecture, including geometry (macroarchitecture) and microarchitecture, and (b) material properties, including mineralization and collagen cross-links, which in turn are influenced by bone turnover as well as by accumulation of microdamage and microstructural discontinuities such as microporosity and lamellar boundaries [9]. Several factors have been claimed to affect bone quality (and mass) in DM, mainly by reducing bone turnover [10]. The advanced glycation endproducts (AGEs) cause the formation of collagen cross-links, which negatively correlate with material properties, in addition to adversely affecting bone metabolism via receptor-mediated mechanisms [11]. Moreover, the long-term, microvascular, and macrovascular complications have been hypothesized to contribute to impaired bone turnover in T1DM and T2DM patients, consistent with the role of vessels [12] and nerves [13] in bone metabolism, and also to favor falls and injury in a fall [14]. In fact, diabetic peripheral neuropathy (DPN) is also an independent risk factor for reduced BMD in the affected limbs [15] and increases the risk of falls by affecting balance and gait [16], thus leading to increased fracture risk [17]. Likewise, the reduction of muscle strength, mass, and quality associated with DM [18] cause not only reduced mechanical strain on the skeleton resulting in reduced bone mass, according to the mechanostat theory [19], but also poor physical performance and reduced mobility favoring falls [20]. In contrast, the impact of hyperinsulinemia and increased body mass index (BMI) associated with T2DM is controversial. On the one hand, they may favor bone formation through direct anabolic effects [21] and increased mechanical load [22], respectively. On the other hand, the low-grade inflammation accompanying insulin resistance can exert detrimental effects on the skeleton [23] and insulin resistance may suppress bone turnover independently of adiposity [24].

Quantitative ultrasound (QUS), which is performed mainly at the heel, provides an estimate of BMD, thus reflecting bone mass [25]. Assessment of estimated BMD (eBMD) by QUS confirmed the increase in BMD detected by dual-energy X-ray absorptiometry (DXA) in patients with T2DM [26]. In older women with T2DM, QUS predicted fracture risk better than DXA [26], though, just like DXA-derived BMD, it failed to identify T2DM subjects with vertebral fractures [27]. In addition, QUS evaluates parameters of bone quality, including microarchitecture and material properties [28, 29].

This study was aimed at assessing the correlates of calcaneal QUS parameters in subjects with T1DM and T2DM, encompassing various degrees of micro- and macrovascular complications and a wide-range of peripheral nerve function.

## 2. Subjects and Methods

### 2.1. Patients

In this cross-sectional analysis, we used the data collected at the baseline visit for the Study on the Assessment of Determinants of Muscle and Bone Strength Abnormalities in Diabetes (SAMBA), an observational, prospective cohort study on the independent predictors of loss of muscle and bone strength in patients with T1DM and T2DM. The study was conducted in accordance with the 1964 Declaration of Helsinki and its later amendments. The research protocol was approved by the locally appointed ethics committee, and participants gave written informed consent.

The SAMBA cohort included 400 patients, 80 with T1DM and 320 with T2DM, who attended consecutively an outpatient diabetes clinics for the screening of complications in years 2008–2011. Patients who were unable to correctly perform the test procedures were excluded from the study [30].

### 2.2. Measurements

#### 2.2.1. Calcaneal QUS

QUS measurements were performed at the heel using the Sahara® Clinical Bone Sonometer (Hologic Inc., Waltham, MA, USA).

Broadband ultrasound attenuation (BUA; in dB/MHz) and speed of sound (SOS; in m/s) were measured, and the quantitative ultrasound index (QUI) or stiffness index (SI) was calculated from them and expressed as absolute values and T- and Z-scores. In turn, eBMD (g/cm^2^) was derived from QUI [25, 26] and expressed as absolute values and T- and Z-scores.

#### 2.2.2. Cardiovascular Risk Factors

All patients underwent a structured interview in order to collect the following information: demographics, lifestyle habits, known DM duration, and current treatments.

Physical activity (PA) level was assessed using the Minnesota Leisure-Time PA questionnaire [30]. Patients were then classified as sedentary (i.e., ≥8 hours/day spent in any waking behavior characterized by an energy expenditure ≤ 1.5 metabolic equivalents (METs) while in a sitting or reclining posture), physically inactive (i.e., not engaged in any physical activity during the week), or physically active (i.e., low PA [<3.5 METs·h^−1^·week^−1^], moderate PA [3.5–10 METs·h^−1^·week^−1^], or high PA [>10 METs·h^−1^·week^−1^]). Cardiorespiratory fitness was assessed by measuring maximal oxygen consumption (VO_2max_) at the treadmill, using a modified Balke and Ware protocol, with direct measurement of oxygen consumption using the gas exchange analyzer (FitMate, Cosmed, Rome, Italy). Muscle strength was assessed in isometric conditions by means of a Digimax electronic dynamometer (Mechatronic GmbH, Germany), as previously described [30].

BMI was calculated from body weight and height, waist circumference was measured at the umbilicus, and fat mass and fat-free mass were assessed by body impedance (Tanita BF664, Vernon Hills, IL, USA). Blood pressure (BP) was measured with a mercury sphygmomanometer with the patients seated with the arm at the heart level. Hypertension was defined by systolic BP ≥ 140 mmHg and/or diastolic BP ≥ 90 and/or antihypertensive treatment.

Hemoglobin- (Hb-) A_1c_ was assessed by a DCCT-aligned high-performance liquid chromatography method (Adams TMA1C HA-8160, Menarini Diagnostics, Florence, Italy). Fasting glucose, triglycerides, total and HDL cholesterol, nitrogen, and uric acid were measured by standard analytical methods using the VITROS 5.1 FS Chemistry System (Ortho-Clinical Diagnostics Inc., Raritan, NJ, USA), and LDL cholesterol was calculated by the Friedewald formula.

#### 2.2.3. Complications

Prevalent cardiovascular disease (CVD) was assessed from medical history by recording previous documented major acute CVD events, including myocardial infarction, stroke, foot ulcer, gangrene and amputation, and revascularization procedures [31]. In addition, carotid intima-media thickness (IMT) and ankle-brachial index (ABI) were assessed as surrogate measures of diabetic macroangiopathy by color-coded duplex sonography (Agilent HP ImagePoint HX, Hewlett Packard, Rome, Italy) and a mercury sphygmomanometer plus a handheld continuous wave Doppler device (Super Doppler 2, Huntleigh Healthcare, Lewis Center, OH), respectively.

Diabetic nephropathy was evaluated by assessing serum creatinine and albuminuria. Serum creatinine was measured by the modified Jaffe method, and estimated glomerular filtration rate (eGFR) was calculated by the chronic kidney disease epidemiology collaboration (CKD-EPI) equation (http://www.qxmd.com/calculate-online/nephrology/ckd-epi-egfr). Albuminuria was assessed as albumin : creatinine ratio (ACR) by measuring albumin and creatinine concentration by immunonephelometry and the modified Jaffe method, respectively, in first-morning, urine samples. Patients were then assigned to one of the following categories of eGFR (ml/min/1.73 m^2^): 1 (≥90), 2 (60–89), 3a (45–59), 3b (30–44), 4 (15–29), and 5 (<15), and albuminuria (mg/g): normoalbuminuria (<30), including normal (<10) and low (<10–29) albuminuria, microalbuminuria (30–299), and macroalbuminuria (≥300) [32]. As previously reported [33], patients were classified according to the National Kidney Foundation's Kidney Disease Outcomes Quality Initiative as having no CKD, albuminuria alone (stages 1-2 CKD), reduced eGFR alone (stage ≥ 3 CKD without albuminuria), or both (stage ≥ 3 CKD with albuminuria).

Diabetic retinopathy (DR) was evaluated by fundus examination in mydriasis. Patients were classified into the following categories: absent DR, mild, moderate or severe nonproliferative DR, proliferative DR, or maculopathy. For further analysis, patients with mild or moderate nonproliferative DR were classified as having nonadvanced DR, whereas those with severe nonproliferative DR, proliferative DR, maculopathy, or blindness were grouped into the advanced, sight-threatening DR category [34].

Definition of DPN was based on symptoms, signs, and neuroelectrophysiological abnormalities according to the Toronto Diabetic Neuropathy Expert Group and further classified in classical symmetrical polyneuropathy, focal or multifocal neuropathy, or DM-associated neuropathy such as chronic inflammatory demyelinating polyneuropathy [35]. Symptoms and signs of DPN were assessed using the Michigan neuropathy screening instrument (MNSI) comprising a 15-item self-administered questionnaire and a lower extremity examination that includes foot inspection, assessment of vibration perception threshold (VPT) at hallux and ankle reflexes, and monofilament testing (http://www.med.umich.edu/borc/profs/documents/svi/MNSI_patient.pdf). The VPT in the left and right malleolus and hallux was assessed by the use of both a diapason and a biothesiometer (Horwell, Nottingham, UK). The distal latencies, amplitudes, and conduction velocities of the peroneal motor nerve (PMN) and sural sensory nerve (SSN) were measured bilaterally by electromyography (EMG), using a Medelec MS 928 Neurostar (Oxford Instruments Medical, Old Woking, UK), and results were compared with age-related reference values [36]. Patients were then stratified for severity of DPN using an arbitrary score calculated as the sum of the individual scores for symptoms (0 to 13, based on the MNSI questionnaire, not considering answers to questions 4 and 10), signs (0 to 10, based on the MNSI lower extremity examination), and EMG data (0 to 12, based on distal latencies, amplitudes, and conduction velocities of sural and peroneal nerves of the two legs).

Autonomic function was assessed by resting heart rate (HR), as a surrogate index, and cardiovascular autonomic reflex tests (CARTs) [37], that is, HR response to deep breathing (beat-to-beat variation and expiration-to-inspiration (EI) ratio), cough test (CT ratio), and standing (30 : 15 ratio), and systolic BP response to standing (supine-standing BP). According to age-related reference values [38], each test was scored as follows: 0 = normal; 1 = borderline, and 2 = abnormal. Subjects were then stratified for cardiac autonomic neuropathy (CAN) based on the sum of the scores assigned to each CART (CAN score).

Maximal voluntary contractions (MVCs) were performed at the shoulder press (Technogym, Gambettola, Italy) along the sagittal plan, with a 45° and 90° angle at the elbow and between the upper arm and the trunk, respectively, for the upper body, and at the leg extension machine (Technogym), with a 90° angle at the knee and the hip, for the lower body [30].

### 2.3. Statistical Analysis

Data are expressed as mean ± SD, for continuous variables, and number of cases and percentages for categorical variables. Patients were stratified by age, gender, type of diabetes, PA level, complications, and treatments, and QUS values were compared using the following statistical tests: the Student *t*-test for parametric or the corresponding Mann–Whitney *U* test for nonparametric continuous variables and the *χ*^2^ test for categorical variables.

Univariate analysis of correlations between QUS parameters and the variables tested was performed using Spearman's rho. Then, linear regression analyses with stepwise variable selection were applied to assess independent correlates of QUS parameters. Covariates were age, gender, PA level, type and duration of DM, HbA_1c_, BMI (or fat mass and fat-free mass or height and weight), muscle strength, and Vo_2max_ plus surrogate measures of complications (model 1) or the presence/absence of CVD, DR and CKD and DPN and CAN scores (model 2). The analyses were conducted both in the whole cohort and separately in T1DM and T2DM patients.

## 3. Results

The clinical characteristics of study subjects are reported in Table 1. Males were more frequently former or current smokers and physically active and had higher VO_2max_ (only T2DM), muscle strength, fat-free mass, waist circumference, and lower BMI, fat mass, and HDL cholesterol, as compared with females. While no gender difference was observed in the prevalence of nephropathy, retinopathy, and neuropathy (though there was a significantly different distribution across CKD phenotypes and categories of somatic neuropathy), CVD was more frequent in males than in females.

As previously reported [30], patients with T2DM from the SAMBA cohort had older age, shorter DM duration, lower PA level, VO_2max_, and upper and lower body strength, as compared with subjects with T1DM. In addition, T2DM patients had lower HbA_1c_, total and HDL cholesterol, eGFR, and ABI, and higher BMI, fat mass, fat-free mass, waist circumference, triglycerides, systolic and diastolic BP, uric acid, nitrogen, ACR, and IMT. Prevalence of CVD and CKD was higher in T2DM patients, whereas retinopathy and somatic neuropathy were more frequent in T1DM subjects (Supplementary Table 1 available online at https://doi.org/10.1155/2017/4749619).

All QUS parameters were higher in males than in females but did not differ between T1DM and T2DM patients, except for QUI- and eBMD-derived Z-scores, which were similar in women and men and lower in individuals with T1DM than in those with T2DM (Table 2). All QUS parameters, except SOS, decreased significantly with age quartiles (not shown), whereas they increased significantly when stratified according to BMI categories (Supplementary Table 2). In addition, there was a nonsignificant trend towards an increase in BUA, SOS, QUI, and eBMD values with increasing ACR and a significant decrease with decreasing eGFR and increasing severity of DR, DPN, and CAN, but not CVD (data not shown).

At univariate analysis, QUS parameters were strongly associated between each other and with upper and lower body strength, cardiorespiratory fitness, BMI, fat-free mass, waist circumference, triglycerides (except SOS), uric acid, and, inversely, age and HDL cholesterol, but not total and LDL cholesterol, BP, and nitrogen, whereas HbA_1c_ correlated with SOS and DM duration with BUA and QUI only. Among measures of complications, eGFR, albuminuria, ABI, PMN amplitude, SSN distal latency and amplitude, and HR response to cough test and standing correlated with some or all QUS parameters (Table 3). When patients were analyzed separately according to the type of DM, QUS parameters were found to correlate significantly with age, muscle strength, fat-free mass, eGFR, SSN amplitude, and HR response to cough test and standing in T1DM patients and with age, muscle strength, BMI, fat-free mass, waist circumference, HbA_1c_, triglycerides, HDL cholesterol, uric acid, serum creatinine, albuminuria, ABI, SSN amplitude, and HR response to standing, in T2DM patients (not shown).

Multivariate analysis revealed that BUA, SOS, QUI, and eBMD were independently associated with age, male gender, BMI, HbA_1c_ (for SOS and eBMD only), and the following somatic and autonomic nerve function parameters (model 1): PMN and SSN amplitude, VPT hallux (for BUA and eBMD only), HR response to cough test (CT ratio, for eBMD only) and standing (30 : 15, for QUI and eBMD only), and, inversely, resting HR (for SOS and eBMD only). Conversely, the type and duration of DM, muscle strength, Vo_2max_, eGFR, carotid IMT, ABI, and CARTs did not enter the model. In patients with T1DM, age, male gender, BMI, PMN and SSN amplitude, VTP allux, and HR response to cough test and standing (for eBMD only), but not HbA_1c_ and resting HR, were independently associated with QUS measures. In individuals with T2DM, correlates of QUS parameters were similar, except for HbA_1c_ and resting HR, which were significantly associated with QUS measures, VPT hallux and HR response to standing, which were not, and HR response to cough test, which was independently associated with SOS instead of eBMD (Table 4). When fat mass and fat-free mass were included in the model in place of BMI, only the former remained as an independent correlate of QUS parameters, either in the whole cohort or in individuals with T1DM or T2DM (not shown). When substituting height and weight for BMI, only weight was an independent correlate of QUS measures (not shown). The presence/absence of CVD, DR and CKD and DPN and CAN scores, when substituted for individual measures of complications, did not enter the model, except for DPN score when the analysis was conducted only in patients with T1DM (model 2, data not shown).

## 4. Discussion

This study provides new information on the correlates of QUS parameters in subjects with DM by showing an independent association with age, male gender, HbA_1c_, BMI, and several measures of complications, particularly of DPN and CAN.

While, as expected, values of BUA, SOS, QUI, and eBMD were higher in males than in females and decreased with increasing age; they were similar in patients with T1DM and T2DM. This somewhat surprising finding might be due to the fact that T2DM subjects, though older, had shorter DM duration than those with T1DM, in addition to reflecting the reduced bone mass observed in T1DM, but not T2DM individuals [2]. Also, correlates of QUS parameters were quite similar in patients with T1DM and T2DM, though there were a few differences (e.g., weaker or no association with BMI and HbA_1c_ and stronger association with surrogate measures of peripheral nerve dysfunction in T1DM) which might reflect the differences in bone microarchitecture detected in T1DM (involvement of both trabecular and cortical compartments) [39] versus T2DM (alteration of the cortical compartment only) [40].

The strong association between BMI and QUS parameters is consistent with a large body of evidence showing that DXA-derived BMD is positively related to BMI in the general population [41] and in diabetic patients [2, 42], an effect likely mediated by mechanical loading on bone [22], and inversely related to fracture risk [43], though this relation varies according to the fracture site [44]. Indeed, the results of a large meta-analysis have indicated that the relationship between BMI and fracture risk is significantly modified by the interaction between BMI and BMD [45] and a recent study has found that the majority of BMI effect on fracture risk is not direct but rather mediated by femoral neck BMD [46]. Our findings are also consistent with the correlation between BMI and QUS parameters observed in previous reports [47–49], though other studies failed to detect an association of SOS [50] or all three QUS measures [51] with BMI.

The observation that QUS parameters were positively related also to waist circumference, fat mass, triglycerides and, inversely, HDL cholesterol, but not mass free-mass (except than in univariate analysis), seems to suggest that adiposity, rather than lean mass, is protective for the skeleton. Though fat mass and lean mass both cause mechanical loading [52], the relative effect of these two determinants of body composition on BMD still remains controversial [53]. Our results seem to be at odds with several studies showing that lean mass, but not fat mass, is positively associated with BMD in men [54], women [55], and both [56]. However, a recent meta-analysis showed that the overall impact of lean mass on femoral neck BMD is higher than that of fat mass and that this is the case in men and premenopausal women, whereas in postmenopausal women the effects of lean mass and fat mass are similar [57]. In addition, our findings do not support a detrimental effect of adiposity on bone health through the associated inflammation [23], though inflammatory markers were not assessed in our study.

Another important observation of this study is the association of QUS parameters with measures of peripheral nerve dysfunction, both somatic and autonomic, including PMN and SSN amplitude, VPT, HR response to cough test and standing, and resting HR. Though the latter parameter is not a specific index of CAN, association with QUS measures was independent of several factors which are known to influence HR, such as BMI, PA level, and VO_2max_. The finding that QUS parameters were associated with peripheral nerve function is in agreement with a previous report from the Health, Aging, and Body Composition Study, showing that peripheral nerve dysfunction was related to lower hip BMD and calcaneal QUS in older adults [58]. It is also in keeping with the higher prevalence of neuropathy (52% versus 22%) in subjects with low versus normal BMD [59] and with the independent association of neuropathy with reduced BMD in the affected limbs as well as elsewhere [15] in patients with T1DM. Thus, our data strongly support the concept that DPN may contribute to bone abnormalities and increased fracturative risk in diabetes.

Strengths of the study are the numerous risk factors and measures of complications considered as potential correlates of QUS parameters. Limitations include the cross-sectional design of the study and the relatively low number of patients with T1DM. Moreover, our work might be limited by the lack of a control group, though the comparison with healthy subjects was beyond the scope of our investigation, which was aimed at identifying QUS correlates in diabetic subjects with various degrees of complications.

## 5. Conclusions

In conclusion, our data indicate that peripheral nerve dysfunction, both somatic and autonomic, is associated with worse QUS parameters, thus suggesting that this complication may contribute to increased fracture risk in diabetes, possibly by impairing bone quality. Conversely, the positive relation of QUS measures with adiposity needs further investigation.

## Supplementary Material

Supplemental Table 1. Prevalence of complications according to type of DM. Supplemental Table 2. QUS values of study subjects according to the BMI category. The Study on the Assessment of Determinants of Muscle and Bone Strength Abnormalities in Diabetes (SAMBA) Investigators.

## Figures and Tables

**Table 1:** Clinical characteristics of study subjects, overall and by gender.

	All	Females	Males	*P*
*N* (%)	400 (100.0)	172 (43.0)	228 (57.0)	
T1DM, *n* (%)	80 (20.0)	42 (24.4)	38 (16.7)	0.055
Age, years	61.9 ± 13.6	63.0 ± 13.8	61.1 ± 13.4	0.155
Smoking, *n* (%)				<0.001
Never	237 (59.2)	132 (76.7)	105 (46.1)	
Former	105 (26.3)	22 (12.8)	83 (36.4)	
Current	58 (14.5)	18 (10.5)	40 (17.5)	
Alcohol intake, *n* (%)				<0.001
No	220 (55.0)	133 (77.3)	87 (38.2)	
Low	130 (32.5)	39 (22.7)	91 (39.9)	
Moderate	42 (10.5)	0 (0.0)	42 (18.4)	
High	8 (2.0)	0 (0)	8 (3.5)	
PA, *n* (%)				<0.001
Sedentary	73 (18.2)	40 (23.3)	33 (14.5)	
Inactive	130 (32.5)	69 (40.1)	61 (26.7)	
Active				
Low PA	84 (21.0)	31 (18.0)	53 (23.3)	
Moderate PA	77 (19.3)	23 (13.4)	54 (23.7)	
High PA	36 (9.0)	9 (5.2)	27 (11.8)	
Upper body muscle strength, *N*	221.7 ± 92.7	157.3 ± 49.1	270.1 ± 88.2	<0.001
Lower body muscle strength, *N*	135.5 ± 62.8	95.7 ± 39.4	164.8 ± 60.7	<0.001
VO_2max_, ml/min/kg	35.3 ± 12.1	33.3 ± 12.6	36.0 ± 11.8	0.182
Family history of DM, *n* (%)	173 (43.3)	71 (41.3)	102 (44.7)	0.490
Duration of DM, years	15.6 ± 10.0	16.7 ± 10.3	14.7 ± 9.7	0.048
HbA_1c_, %	7.15 ± 1.44	7.24 ± 1.41	7.09 ± 1.46	0.313
Glucose, mmol/l	8.59 ± 3.51	8.39 ± 3.35	8.74 ± 3.63	0.323
BMI, kg/m^2^	28.8 ± 5.7	29.57 ± 6.83	28.23 ± 4.62	0.020
Fat mass, %	29.6 ± 10.0	37.00 ± 7.35	24.09 ± 7.86	<0.001
Fat free mass, kg	55.2 ± 11.2	45.50 ± 6.66	62.44 ± 7.95	<0.001
Waist circumference, cm	97.5 ± 13.9	94.16 ± 14.57	100.07 ± 12.90	<0.001
Triglycerides, mmol/l	1.65 ± 1.06	1.90 ± 0.63	1.99 ± 0.70	0.016
Total cholesterol, mmol/l	4.77 ± 0.99	4.98 ± 0.95	4.61 ± 0.99	<0.001
HDL cholesterol, mmol/l	1.28 ± 0.37	1.43 ± 0.37	1.16 ± 0.32	<0.001
LDL cholesterol, mmol/l	2.82 ± 0.98	2.91 ± 0.90	2.75 ± 1.04	0.097
SBP, mmHg	135.0 ± 17.2	137.35 ± 18.58	133.22 ± 15.81	0.017
DBP, mmHg	78.7 ± 8.9	78.95 ± 9.77	78.51 ± 8.23	0.622
Carotid IMT, mm	1.01 ± 0.16	0.98 ± 0.17	1.02 ± 0.16	0.025
ABI	0.89 ± 0.15	0.87 ± 0.15	0.91 ± 0.15	0.019
Uric acid, *μ*mol/l	294.1 ± 99.7	267.1 ± 101.6	312.7 ± 93.9	<0.001
Plasma nitrogen, mmol/l	16.9 ± 7.1	17.2 ± 7.7	16.6 ± 6.5	0.448
Serum creatinine, *μ*mol/l	99.3 ± 35.1	89.7 ± 30.5	106.6 ± 36.6	<0.001
eGFR, ml/min/1.73 m^2^	68.3 ± 20.2	65.1 ± 20.0	70.7 ± 20.1	0.006
ACR, mg/g	58.6 ± 65.5	56.1 ± 79.9	60.5 ± 52.2	0.507
PMN distal latency, m/s	4.98 ± 0.65	4.80 ± 0.58	5.12 ± 0.67	<0.001
PMN amplitude, mV	3.57 ± 1.35	3.62 ± 1.25	3.53 ± 1.42	0.480
PMN conduction velocity, m/s	44.8 ± 4.3	45.6 ± 3.7	44.2 ± 4.6	0.002
SSN distal latency, m/s	3.29 ± 0.72	3.19 ± 0.66	3.36 ± 0.75	0.018
SSN amplitude, mV	11.6 ± 8.0	11.9 ± 8.2	11.4 ± 8.0	0.560
SSN conduction velocity, m/s	39.5 ± 11.4	40.0 ± 11.5	39.2 ± 11.3	0.496
VPT malleolus, mV	25.9 ± 13.9	25.0 ± 13.7	26.6 ± 14.1	0.262
VPT hallux, mV	20.8 ± 13.7	20.0 ± 13.3	21.5 ± 14.0	0.296
Resting HR, beats/min	66.9 ± 10.1	69.0 ± 9.3	65.4 ± 10.5	0.001
HR response to deep breathing, EI ratio	1.24 ± 0.16	1.24 ± 0.15	1.24 ± 0.16	0.818
HR response to cough test, CT ratio	1.22 ± 0.15	1.20 ± 0.16	1.24 ± 0.15	0.027
HR response to standing, 30 : 15 ratio	1.14 ± 0.13	1.13 ± 0.12	1.15 ± 0.13	0.105
SBP response to standing, Δ(mmHg)	4.92 ± 6.48	5.39 ± 6.81	4.56 ± 6.22	0.214
CVD, *n* (%)	51 (12.8)	11 (6.4)	40 (17.5)	0.001
CKD, *n* (%)				<0.0001
No	237 (59.3)	100 (58.1)	137 (60.1)	
Albuminuria alone	54 (13.5)	13 (7.6)	41 (18.0)	
Reduced eGFR alone	68 (17.0)	42 (24.4)	26 (11.4)	
Both	41 (10.2)	17 (9.9)	24 (10.5)	
DR, *n* (%)				0.317
No	262 (65.5)	107 (62.2)	155 (68.0)	
Nonadvanced	63 (15.7)	27 (15.7)	36 (15.8)	
Advanced	75 (18.8)	38 (22.1)	37 (16.2)	
DPN, *n* (%)				<0.0001
No	117 (29.2)	51 (29.6)	66 (29.0)	
Polyneuropathy				
Possible	53 (13.2)	28 (16.3)	25 (11.0)	
Probable	19 (4.8)	16 (9.3)	3 (1.3)	
Confirmed	153 (38.2)	68 (39.5)	85 (37.3)	
Subclinical	35 (8.8)	8 (4.7)	27 (11.8)	
Focal or multifocal neuropathy	15 (3.8)	1 (0.6)	14 (6.1)	
Diabetes-associated neuropathy	8 (2.0)	0 (0.0)	8 (3.5)	
CAN, *n* (%)				0.817
No	267 (66.8)	114 (66.3)	153 (67.1)	
Borderline	40 (10.0)	15 (8.7)	25 (11.0)	
Yes	93 (23.2)	43 (25.0)	50 (21.9)	

T1DM = type 1 DM; PA = physical activity; VO_2max_ = maximal oxygen consumption; HbA_1c_ = hemoglobin A_1c_; BMI = body mass index; SBP = systolic blood pressure; DBP = diastolic blood pressure; IMT = intima-media thickness; ABI = ankle-brachial index; eGFR = estimated glomerular filtration rate; ACR = albumin : creatinine ratio; PMN peroneal motor nerve; SSN = sural sensory nerve; VPT = vibration perception threshold; HR = heart rate; EI = expiration-to-inspiration; CT = cough test; CVD = cardiovascular disease; CKD = chronic kidney disease; DPN = diabetic peripheral neuropathy; CAN = cardiac autonomic neuropathy.

**Table 2:** QUS values of study subjects, overall and by gender and type of DM.

QUS parameters	All	Females	Males	*P*	T1DM	T2DM	*P*
BUA, dB/MHz	71.6 ± 22.1	62.6 ± 22.1	78.3 ± 19.5	<0.0001	71.5 ± 24.9	71.6 ± 21.4	0.964
SOS, m/s	1541 ± 80	1527.3 ± 114.5	1551.8 ± 33.4	0.002	1548.6 ± 38.9	1539.3 ± 87.4	0.354
QUI	92.0 ± 22.9	84.9 ± 23.1	97.3 ± 21.3	<0.0001	93.3 ± 25.8	91.6 ± 22.2	0.561
T-score	−0.80 ± 1.33	−1.06 ± 1.38	−0.61 ± 1.27	0.001	−0.69 ± 1.53	−0.83 ± 1.28	0.376
Z-score	0.19 ± 1.28	0.25 ± 1.36	0.16 ± 1.24	0.476	−0.10 ± 1.46	0.27 ± 1.23	0.028
eBMD, g/cm^2^	0.50 ± 0.15	0.45 ± 0.16	0.54 ± 0.13	<0.0001	0.50 ± 0.19	0.50 ± 0.14	0.915
T-score	−0.83 ± 1.32	−1.09 ± 1.38	−0.63 ± 1.25	<0.0001	−0.70 ± 1.53	−0.86 ± 1.27	0.324
Z-score	0.20 ± 1.28	0.23 ± 1.37	0.18 ± 1.23	0.682	−0.08 ± 1.45	0.27 ± 1.23	0.033

QUS = quantitative ultrasound; DM = diabetes mellitus; T1DM = type 1 DM; T2DM = type 2 DM; BUA = broadband ultrasound attenuation; SOS = speed of sound; QUI = quantitative ultrasound index; eBMD = estimated bone mineral density.

**Table 3:** Univariate correlations between QUS parameters and CVD risk factors and measures of complications (Spearman's rho).

Variable	BUA		SOS		QUI		eBMD	
	*r*	*P*	*r*	*P*	*r*	*P*	*r*	*P*
Age	−0.176	<0.0001	−0.261	<0.0001	−0.232	<.00001	−0.225	<0.0001
Upper body strength	0.287	<0.0001	0.234	<0.0001	0.261	<0.0001	0.259	<0.0001
Lower body strength	0.288	<0.0001	0.252	<0.0001	0.271	<0.0001	0.273	<0.0001
Vo_2max_	0.289	<0.0001	0.242	<0.0001	0.258	<0.0001	0.262	<0.0001
DM duration	−0.129	0.010	−0.070	0.162	−0.100	0.047	−0.092	0.068
HbA_1c_	0.055	0.273	0.104	0.038	0.087	0.083	0.089	0.075
Glucose	0.047	0.349	0.048	0.341	0.051	0.310	0.051	0.314
BMI	0.194	<0.0001	0.129	0.010	0.174	<0.0001	0.174	<0.0001
Fat mass	−0.087	0.084	−0.051	0.315	−0.051	0.307	−0.060	0.229
Fat-free mass	0.386	<0.0001	0.278	<0.0001	0.328	<0.0001	0.336	<0.0001
Waist circumference	0.248	<0.0001	0.160	0.001	0.212	<0.0001	0.217	<0.0001
Triglycerides	0.162	0.001	0.094	0.060	0.133	0.008	0.137	0.006
Total cholesterol	−0.053	0.292	−0.019	0.713	−0.027	0.597	−0.028	0.578
HDL cholesterol	−0.251	<0.0001	−0.182	<0.0001	−0.217	<0.0001	−0.217	<0.0001
LDL cholesterol	−0.003	0.947	0.026	0.600	0.019	0.704	0.017	0.736
SBP	−0.055	0.274	−0.090	0.073	−0.072	0.151	−0.069	0.169
DBP	0.001	0.984	−0.013	0.793	0.001	0.981	0.002	0.975
Carotid IMT	0.030	0.548	−0.030	0.556	−0.005	0.915	−0.003	0.958
ABI	0.084	0.093	0.107	0.034	0.097	0.052	0.093	0.063
Uric acid	0.166	0.001	0.112	0.026	0.139	0.005	0.132	0.008
Plasma nitrogen	−0.056	0.263	−0.078	0.122	−0.085	0.088	−0.072	0.150
Serum creatinine	0.077	0.126	0.092	0.069	0.086	0.088	0.088	0.082
eGFR	0.070	0.161	0.103	0.041	0.102	0.041	0.096	0.057
ACR	0.095	0.057	0.115	0.022	0.107	0.033	0.116	0.021
PMN distal latency	0.069	0.167	0.062	0.215	0.063	0.209	0.075	0.134
PMN amplitude	0.072	0.150	0.107	0.034	0.101	0.045	0.091	0.068
PMN conduction velocity	0.015	0.771	0.021	0.684	0.032	0.526	0.019	0.710
SSN distal latency	0.120	0.017	0.109	0.029	0.113	0.024	0.113	0.025
SSN amplitude	0.096	0.055	0.133	0.008	0.133	0.008	0.120	0.016
SSN conduction velocity	0.034	0.494	0.053	0.294	0.060	0.235	0.046	0.358
VPT malleolus	−0.047	0.346	−0.073	0.144	−0.062	0.218	−0.050	0.318
VPT hallux	−0.045	0.365	−0.071	0.156	−0.061	0.221	−0.050	0.319
Resting HR	−0.080	0.109	−0.085	0.090	−0.089	0.075	−0.092	0.067
EI ratio	0.056	0.273	0.069	0.175	0.071	0.165	0.069	0.178
CT ratio	0.099	0.052	0.123	0.015	0.119	0.019	0.123	0.015
30 : 15ratio	0.114	0.025	0.181	<0.0001	0.161	0.001	0.157	0.002
Systolic BP fall	0.031	0.546	−0.011	0.822	0.005	0.914	0.004	0.938
BUA	—	—	0.850	<0.0001	0.935	<0.0001	0.930	<0.0001
SOS	0.850	<0.0001	—	—	0.973	<0.0001	0.973	<0.0001
QUI	0.935	<0.0001	0.973	<0.0001	—	—	0.995	<0.0001
eBMD	0.930	<0.0001	0.973	<0.0001	0.995	<0.0001	—	—

QUS = quantitative ultrasound; CVD = cardiovascular disease; BUA = broadband ultrasound attenuation; SOS = speed of sound; QUI = quantitative ultrasound index; eBMD = estimated bone mineral density; DM = diabetes mellitus; VO_2max_ = maximal oxygen consumption; HbA_1c_ = hemoglobin A_1c_; BMI = body mass index; SBP = systolic blood pressure; DBP = diastolic blood pressure; IMT = intima-media thickness; ABI = ankle-brachial index; eGFR = estimated glomerular filtration rate; ACR = albumin : creatinine ratio; PMN peroneal motor nerve; SSN = sural sensory nerve; VPT = vibration perception threshold; HR = heart rate; EI = expiration : inspiration; CT = cough test.

**Table 4:** Independent correlates of QUS parameters by multiple regression analysis with stepwise variable selection, in the whole cohort and in patients with T1DM and T2DM separately.

Variable	BUA		SOS		QUI		eBMD	
	Beta	*P*	Beta	*P*	Beta	*P*	Beta	*P*
Whole cohort								
Age	−0.284	0.001	−0.344	<0.0001	−0.342	<0.0001	−0.236	0.001
Male gender	0.356	<0.0001	0.191	0.011	0.245	0.001	0.244	0.001
HbA_1c_	—	—	0.169	0.047	—	—	0.148	0.043
BMI	0.290	<0.0001	0.334	<0.0001	0.355	<0.0001	0.299	<0.0001
PMN amplitude	0.153	0.035	0.181	0.018	0.160	0.031	0.190	0.012
SSN amplitude	0.228	0.017	0.168	0.028	0.185	0.016	0.180	0.018
VPT hallux	—	—	0.329	0.009	0.259	0.015	—	—
Resting HR	—	—	−0.192	0.012	—	—	−0.196	0.010
HR response to cough test (CT ratio)							0.143	0.050
HR response to standing (30 : 15 ratio)	—	—	—	—	0.176	0.022	0.139	0.021
T1DM								
Age	−0.450	0.001	−0.597	<0.0001	−0.462	0.001	−0.319	0.020
Male gender	0.296	0.007	—	—	0.230	0.032	0.258	0.019
HbA_1c_	—	—	—	—	—	—	—	—
BMI	0.371	0.007	—	—	0.288	0.018	0.395	0.002
PMN amplitude	0.679	<0.0001	0.510	0.002	0.547	0.001	0.739	<0.0001
SSN amplitude	0.296	0.041	—	—	0.299	0.042	—	—
VPT hallux	0.502	0.007	0.476	0.023	0.406	0.031	0.410	0.040
Resting HR	—	—	—	—	—	—	—	—
HR response to cough test (CT ratio)							0.251	0.027
HR response to standing (30 : 15 ratio)	—	—	—	—	—	—	0.246	0.024
T2DM								
Age	—	—	—	—	−0.134	0.014	−0.169	0.003
Male gender	0.396	<0.0001	0.218	<0.0001	0.283	<0.0001	0.272	<0.0001
HbA_1c_	0.113	0.029	0.140	0.012	0.136	0.011	0.119	0.030
BMI	0.344	<0.0001	0.392	<0.0001	0.414	<0.0001	0.409	<0.0001
PMN amplitude	0.175	0.048	0.128	0.044	0.205	0.023	0.132	0.034
SSN amplitude	—	—	0.166	0.011	0.215	<0.0001	0.163	0.011
VPT hallux	—	—	—	—	—	—	—	—
Resting HR	—	—	−0.120	0.032	—	—	−0.114	0.037
HR response to cough test (CT ratio)	—	—	0.204	0.019	—	—	—	—
HR response to standing (30 : 15 ratio)	—	—	—	—	—	—	—	—

QUS = quantitative ultrasound; T1DM = type 1 DM; T2DM = type 2 DM; BUA = broadband ultrasound attenuation; SOS = speed of sound; QUI = quantitative ultrasound index; eBMD = estimated bone mineral density; HbA_1c_ = hemoglobin A_1c_; BMI = body mass index; PMN peroneal motor nerve; SSN = sural sensory nerve; VPT = vibration perception threshold; HR = heart rate.

## Abbreviations

DM: Diabetes mellitus
T1DM: Type 1 DM
T2DM: Type 2 DM
BMD: Bone mineral density
AGEs: Advanced glycation endproducts
DPN: Diabetic peripheral neuropathy
BMI: Body mass index
QUS: Quantitative ultrasound
eBMD: Estimated BMD
DXA: Dual-energy X-ray absorptiometry
SAMBA: Study on the Assessment of Determinants of Muscle and Bone Strength Abnormalities in Diabetes
BUA: Broadband ultrasound attenuation
SOS: Speed of sound
QUI: Quantitative ultrasound index
PA: Physical activity
METs: Metabolic equivalents
VO_2max_: Maximal oxygen consumption
BP: Blood pressure
HbA_1c_: Hemoglobin A_1c_
CVD: Cardiovascular disease
IMT: Intima-media thickness
ABI: Ankle-brachial index
eGFR: Estimated glomerular filtration rate
ACR: Albumin : creatinine ratio
CKD: Chronic kidney disease
DR: Diabetic retinopathy
MNSI: Michigan neuropathy screening instrument
VPT: Vibration perception threshold
PMN: Peroneal motor nerve
SSN: Sural sensory nerve
EMG: Electromyography
HR: Heart rate
CARTs: Cardiovascular autonomic reflex tests
EI: Expiration-to-inspiration
CT: Cough test
CAN: Cardiac autonomic neuropathy
MCVs: Maximal voluntary contractions.

## References

[1] Shanbhogue V. V., Mitchell D. M., Rosen C. J., Bouxsein M. L. (2016). Type 2 diabetes and the skeleton: new insights into sweet bones. *The Lancet Diabetes and Endocrinology*.

[2] Vestergaard P. (2007). Discrepancies in bone mineral density and fracture risk in patients with type 1 and type 2 diabetes—a meta-analysis. *Osteoporosis International*.

[3] Hernlund E., Svedbom A., Ivergård M. (2013). Osteoporosis in the European Union: medical management, epidemiology and economic burden. A report prepared in collaboration with the International Osteoporosis Foundation (IOF) and the European Federation of Pharmaceutical Industry Associations (EFPIA). *Archives of Osteoporosis*.

[4] Schwartz A. V., Sellmeyer D. E., Ensrud K. E. (2001). Older women with diabetes have an increased risk of fracture: a prospective study. *The Journal of Clinical Endocrinology and Metabolism*.

[5] Strotmeyer E. S., Cauley J. A., Schwartz A. V. (2005). Nontraumatic fracture risk with diabetes mellitus and impaired fasting glucose in older white and black adults: the health, aging, and body composition study. *Archives of Internal Medicine*.

[6] Napoli N., Strotmeyer E. S., Ensrud K. E. (2014). Fracture risk in diabetic elderly men: the MrOS study. *Diabetologia*.

[7] Bonds D. E., Larson J. C., Schwartz A. V. (2006). Risk of fracture in women with type 2 diabetes: the Women’s health initiative observational study. *The Journal of Clinical Endocrinology and Metabolism*.

[8] Schwartz A. V., Hillier T. A., Sellmeyer D. E. (2002). Older women with diabetes have a higher risk of falls: a prospective study. *Diabetes Care*.

[9] Farr J. N., Khosla S. (2016). Determinants of bone strength and quality in diabetes mellitus in humans. *Bone*.

[10] Hygum K., Starup-Linde J., Harsløf T., Vestergaard P., Langdahl B. L. (2017). Mechanisms in endocrinology: diabetes mellitus, a state of low bone turnover - a systematic review and meta-analysis. *European Journal of Endocrinology*.

[11] Yamamoto M., Sugimoto T. (2016). Advanced glycation end products, diabetes, and bone strength. *Current Osteoporosis Reports*.

[12] Parfitt A. M. (2000). The mechanism of coupling: a role for the vasculature. *Bone*.

[13] Spencer G. J., Hitchcock I. S., Genever P. G. (2004). Emerging neuroskeletal signalling pathways: a review. *FEBS Letters*.

[14] Nelson D. A., Jacober S. J. (2001). Why do older women with diabetes have an increased fracture risk?. *The Journal of Clinical Endocrinology and Metabolism*.

[15] Rix M., Andreassen H., Eskildsen P. (1999). Impact of peripheral neuropathy on bone density in patients with type 1 diabetes. *Diabetes Care*.

[16] Richardson J. K., Hurvitz E. A. (1995). Peripheral neuropathy: a true risk factor for falls. *The Journals of Gerontology. Series A, Biological Sciences and Medical Sciences*.

[17] Melton L. J., Leibson C. L., Achenbach S. J., Therneau T. M., Khosla S. (2008). Fracture risk in type 2 diabetes: update of a population-based study. *Journal of Bone and Mineral Research*.

[18] Park S. W., Goodpaster B. H., Strotmeyer E. S. (2006). Decreased muscle strength and quality in older adults with type 2 diabetes: the health, aging, and body composition study. *Diabetes*.

[19] Frost H. M. (2003). Bone’s mechanostat: a 2003 update. *The Anatomical Record. Part A, Discoveries in Molecular, Cellular, and Evolutionary Biology*.

[20] Volpato S., Leveille S. G., Blaum C., Fried L. P., Guralnik J. M. (2005). Risk factors for falls in older disabled women with diabetes: the women’s health and aging study. *The Journals of Gerontology. Series A, Biological Sciences and Medical Sciences*.

[21] Fulzele K., Riddle R. C., DiGirolamo D. J. (2010). Insulin receptor signaling in osteoblasts regulates postnatal bone acquisition and body composition. *Cell*.

[22] Walsh J. S., Vilaca T. (2017). Obesity, type 2 diabetes and bone in adults. *Calcified Tissue International*.

[23] Greco E. A., Fornari R., Rossi F. (2010). Is obesity protective for osteoporosis? Evaluation of bone mineral density in individuals with high body mass index. *International Journal of Clinical Practice*.

[24] Tonks K. T., White C. P., Center J. R., Samocha-Bonet D., Greenfield J. R., Bone Turnover I. (2017). Suppressed in insulin resistance, independent of adiposity. *The Journal of Clinical Endocrinology and Metabolism*.

[25] Glüer C. C. (1997). Quantitative ultrasound techniques for the assessment of osteoporosis: expert agreement on current status. The international quantitative ultrasound consensus group. *Journal of Bone and Mineral Research*.

[26] Patel S., Hyer S., Tweed K. (2008). Risk factors for fractures and falls in older women with type 2 diabetes mellitus. *Calcified Tissue International*.

[27] Yamaguchi T., Yamamoto M., Kanazawa I. (2011). Quantitative ultrasound and vertebral fractures in patients with type 2 diabetes. *Journal of Bone and Mineral Metabolism*.

[28] Padilla F., Jenson F., Bousson V., Peyrin F., Laugier P. (2008). Relationships of trabecular bone structure with quantitative ultrasound parameters: in vitro study on human proximal femur using transmission and backscatter measurements. *Bone*.

[29] Bouxsein M. L., Radloff S. E. (1997). Quantitative ultrasound of the calcaneus reflects the mechanical properties of calcaneal trabecular bone. *Journal of Bone and Mineral Research*.

[30] Balducci S., Sacchetti M., Orlando G. (2014). Correlates of muscle strength in diabetes. The study on the assessment of determinants of muscle and bone strength abnormalities in diabetes (SAMBA). *Nutrition, Metabolism, and Cardiovascular Diseases*.

[31] Solini S., Penno G., Bonora E. (2012). Diverging association of reduced glomerular filtration rate and albuminuria with coronary and noncoronary events in patients with type 2 diabetes: the renal insufficiency and cardiovascular events (RIACE) Italian multicentre study. *Diabetes Care*.

[32] Pugliese G., Solini A., Fondelli C. (2011). Reproducibility of albuminuria in type 2 diabetic subjects. Findings from the renal insufficiency and cardiovascular events (RIACE) study. *Nephrology, Dialysis, Transplantation*.

[33] Penno G., Solini A., Bonora E. (2011). Clinical significance of nonalbuminuric renal impairment in type 2 diabetes. *Journal of Hypertension*.

[34] Penno G., Solini A., Zoppini G. (2012). Rate and determinants of association between advanced retinopathy and chronic kidney disease in patients with type 2 diabetes: the renal insufficiency and cardiovascular events (RIACE) Italian multicenter study. *Diabetes Care*.

[35] Tesfaye S., Boulton A. J., Dyck P. J. (2010). Diabetic neuropathies: update on definitions, diagnostic criteria and estimation of severity (the Toronto Expert Group Meeting 2009). *Diabetes Care*.

[36] Oh S. J., OH S. J. (2002). Normal values for common nerve conduction tests. *Clinical Electromyography: Nerve Conduction Studies*.

[37] Spallone V., Bellavere F., Scionti L. (2011). Recommendations for the use of cardiovascular tests in diagnosing diabetic autonomic neuropathy. *Nutrition, Metabolism, and Cardiovascular Diseases*.

[38] Cardone C., Paiusco P., Marchetti G., Burelli F., Feruglio M., Fedele D. (1990). Cough test to assess cardiovascular autonomic reflexes in diabetes. *Diabetes Care*.

[39] Shanbhogue V. V., Hansen S., Frost M. (2015). Bone geometry, volumetric density, microarchitecture, and estimated bone strength assessed by hr-pqct in adult patients with type 1 diabetes mellitus. *Journal of Bone and Mineral Research*.

[40] Shanbhogue V. V., Hansen S., Frost M. (2016). Compromised cortical bone compartment in type 2 diabetes mellitus patients with microvascular disease. *European Journal of Endocrinology*.

[41] Felson D. T., Zhang Y., Hannan M. T., Anderson J. J. (1993). Effects of weight and body mass index on bone mineral density in men and women: the Framingham study. *Journal of Bone and Mineral Research*.

[42] Ma L., Oei L., Jiang L. (2012). Association between bone mineral density and type 2 diabetes mellitus: a meta-analysis of observational studies. *European Journal of Epidemiology*.

[43] Laet C., Kanis J. A., Odén A. (2005). Body mass index as a predictor of fracture risk: a meta-analysis. *Osteoporosis International*.

[44] Compston J. E., Flahive J., Hosmer D. W. (2014). Relationship of weight, height, and body mass index with fracture risk at different sites in postmenopausal women: the global longitudinal study of osteoporosis in women (GLOW). *Journal of Bone and Mineral Research*.

[45] Johansson H., Kanis J. A., Odén A. (2014). A meta-analysis of the association of fracture risk and body mass index in women. *Journal of Bone and Mineral Research*.

[46] Chan M. Y., Frost S. A., Center J. R., Eisman J. A., Nguyen T. V. (2014). Relationship between body mass index and fracture risk is mediated by bone mineral density. *Journal of Bone and Mineral Research*.

[47] Bulló M., Garcia-Aloy M., Basora J., Covas M. I., Salas-Salvado J. (2011). Bone quantitative ultrasound measurements in relation to the metabolic syndrome and type 2 diabetes mellitus in a cohort of elderly subjects at high risk of cardiovascular disease from the PREDIMED study. *The Journal of Nutrition, Health & Aging*.

[48] Cvijetic S., Pavlovic M., Pasalic D., Dodig S. (2011). Ultrasound bone measurement in an older population with metabolic syndrome. *Aging Clinical and Experimental Research*.

[49] Korpelainen R., Korpelainen J., Heikkinen J., Väänänen K., Keinänen-Kiukaanniemi S. (2006). Lifelong risk factors for osteoporosis and fractures in elderly women with low body mass index—a population-based study. *Bone*.

[50] Steinschneider M., Hagag P., Rapoport M. J., Weiss M. (2003). Discordant effect of body mass index on bone mineral density and speed of sound. *BMC Musculoskeletal Disorders*.

[51] Sosa M., Saavedra P., Jódar E. (2009). Bone mineral density and risk of fractures in aging, obese post-menopausal women with type 2 diabetes. The GIUMO study. *Aging Clinical and Experimental Research*.

[52] Reid I. R. (2010). Fat and bone. *Archives of Biochemistry and Biophysics*.

[53] Gonnelli S., Caffarelli C., Nuti R. (2014). Obesity and fracture risk. *Clinical Cases in Mineral and Bone Metabolism*.

[54] Douchi T., Kuwahata R., Matsuo T., Uto H., Oki T., Nagata Y. (2003). Relative contribution of lean and fat mass component to bone mineral density in males. *Journal of Bone and Mineral Metabolism*.

[55] Beck T. J., Petit M. A., GL W., Leboff M. S., Cauley J. A., Chen Z. (2009). Does obesity really make the femur stronger? BMD, geometry, and fracture incidence in the women’s health initiative-observational study. *Journal of Bone and Mineral Research*.

[56] Leslie W. D., Orwoll E. S., Nielson C. M. (2014). Estimated lean mass and fat mass differentially affect femoral bone density and strength index but are not FRAX independent risk factors for fracture. *Journal of Bone and Mineral Research*.

[57] Ho-Pham L. T., Nguyen U. D., Nguyen T. V. (2014). Association between lean mass, fat mass, and bone mineral density: a meta-analysis. *The Journal of Clinical Endocrinology and Metabolism*.

[58] Strotmeyer E. S., Cauley J. A., Schwartz A. V. (2006). Reduced peripheral nerve function is related to lower hip BMD and calcaneal QUS in older white and black adults: the health, aging, and body composition study. *Journal of Bone and Mineral Research*.

[59] Kayath M. J., Dib S. A., Vieira J. G. (1994). Prevalence and magnitude of osteopenia associated with insulin-dependent diabetes mellitus. *Journal of Diabetes and its Complications*.

