# Supplementary material for: Correlates of Calcaneal Quantitative Ultrasound Parameters in Patients with Diabetes: The Study on the Assessment of Determinants of Muscle and Bone Strength Abnormalities in Diabetes
Source: J Diabetes Res. 2017 Sep 19;2017:4749619. doi: 10.1155/2017/4749619 (PMC5625809; doi:10.1155/2017/4749619)
Supplement: Supplementary file 1 — Supplemental Table 1. Prevalence of complications according to type of DM. Supplemental Table 2. QUS values of study subjects according to the BMI category. The Study on the Assessment of Determinants of Muscle and Bone Strength Abnormalities in Diabetes (SAMBA) Investigators. [file 4749619.f1.doc]

**Supplemental Table 1.** Prevalence of complications according to type of DM.

|  | **All** | **T1DM** | **T2DM** | ***P*** |
| --- | --- | --- | --- | --- |
| **N (%)** | 400 (100.0) | 80 (20.0) | 320 (80.0) |  |
| **CVD, n (%)** | 3 (3.8) | 48 (15.0) | 51 (12.8) | 0.007 |
| **CKD, n (%)** |  |  |  | <0.0001 |
| **No** | 237 (59.2) | 63 (78.8) | 174 (54.4) |  |
| **Albuminuria alone** | 54 (13.5) | 9 (11.3) | 45 (14.1) |  |
| **Reduced eGFR alone** | 68 (17.0) | 7 (8.7) | 61 (19.0) |  |
| **Both** | 41 (10.3) | 1 (1.2) | 40 (12.5) |  |
| **DR, n (%)** |  |  |  | 0.002 |
| **No** | 262 (65.5) | 40 (50.0) | 222 (69.4) |  |
| **Nonadvanced** | 63 (15.7) | 15 (18.7) | 48 (15.0) |  |
| **Advanced** | 75 (18.8) | 25 (31.2) | 50 (15.6) |  |
| **DPN, n (%)** |  |  |  | 0.003 |
| **No** | 117 (29.3) | 33 (41.3) | 84 (26.2) |  |
| **Polyneuropathy** |  |  |  |  |
| **Possible** | 53 (13.3) | 2 (2.5) | 51 (15.9) |  |
| **Probable** | 19 (4.7) | 3 (3.7) | 16 (5.0) |  |
| **Confirmed** | 153 (38.3) | 25 (31.3) | 128 (40.0) |  |
| **Subclinical** | 35 (8.7) | 12 (15.0) | 23 (7.2) |  |
| **Focal or multifocal neuropathy** | 15 (3.7) | 3 (3.7) | 12 (3.8) |  |
| **Diabetes-associated neuropathy** | 8 (2.0) | 2 (2.5) | 6 (1.9) |  |
| **CAN, n (%)** |  |  |  | 0.328 |
| **No** | 267 (66.7) | 59 (73.7) | 208 (65.0) |  |
| **Bordeline** | 40 (10.0) | 6 (7.5) | 34 (10.6) |  |
| **Yes** | 93 (23.3) | 15 (18.8) | 78 (24.4) |  |

DM = diabetes mellitus; T1DM = type 1 DM; T2DM = type 2 DM; CVD = cardiovascular disease; CKD = chronic kidney disease; DPN = diabetic peripheral neuropathy; CAN = cardiac autonomic neuropathy.

**Supplemental Table 2.** QUS values of study subjects according to the BMI category.

| **QUS parameters** | **Normal-weight** | **Overweight** | **Obese, I grade** | **Obese, II grade** | **Obese, III grade** | ***P*** |
| --- | --- | --- | --- | --- | --- | --- |
|  | **(n=101)** | **(n=155)** | **(n=88)** | **(n=38)** | **(n=18)** |  |
| **BUA, dB/MHz** | 67.4±25.8 | 69.7±19.3 | 76.1±19.7 | 76.7±24.2 | 77.6±22.9 | 0.018 |
| **SOS, m/s** | 1,527.3±149.1 | 1,542.5±30.3 | 1,546.9±31.8 | 1,548.4±40.4 | 1,564.5±29.7 | 0.254 |
| **QUI** | 88.5±26.3 | 90.1±19.9 | 94.4±20.6 | 96.9±28.6 | 104.5±18.3 | 0.021 |
| **T-score** | -0.99±1.56 | -0.95±1.14 | -0.66±1.23 | -0.49±1.49 | 0.16±1.20 | 0.002 |
| **Z-score** | -0.14±1.44 | 0.05±1.08 | 0.33±1.12 | 0.70±1.51 | 1.36±1.19 | <0.0001 |
| **eBMD, g/cm^2^** | 0.47±0.19 | 0.49±0.12 | 0.52±0.13 | 0.53±0.16 | 0.58±0.11 | 0.010 |
| **T-score** | -1.01±1.54 | -0.96±1.14 | -0.70±1.19 | -0.56±1.56 | 0.11±1.18 | 0.005 |
| **Z-score** | -0.11±1.42 | 0.07±1.09 | 0.33±1.11 | 0.65±1.56 | 1.26±1.25 | <0.0001 |

QUS = quantitative ultrasound; DM = diabetes mellitus; T1DM = type 1 DM; T2DM = type 2 DM; BUA = broadband ultrasound attenuation; SOS = speed of sound; QUI = quantitative ultrasound index; eBMD = estimated bone mineral density.

**The Study on the Assessment of Determinants of Muscle and Bone Strength Abnormalities in Diabetes (SAMBA) Investigators**

**Health Care Team**

Di Biase Nicolina, MD, Diabetes Unit, Fatebenefratelli San Pietro Hospital, Rome, Italy

Alessandra Bazuro, MD, and Carla Maccora, MD, Diabetes Unit, Sant’Andrea Hospital, Rome, Italy

Patrizia Cardelli, Laboratory of Clinical Chemistry, Sant’Andrea Hospital, Rome, Italy

Eugenio Calandriello, MD, Neurology and Neuro-Pathophysiology Unit, Regina Apostolorum Hospital, Albano, Rome, Italy

Silvia Lai, MD, Nephrology and Dialysis Unit, Policlinico Umberto I, and Department of Clinical Medicine, “La Sapienza” University, Rome, Italy

Fabio Ferranti, MD, Cardiology Unit, G.B. Grassi Hospital, Rome, Italy

**Sport and Exercise Team**

Massimo Sacchetti, PhD, and Giorgio Orlando, PhD, Department of Human Movement and Sport Sciences, ‘‘Foro Italico’’ University, Rome, Italy

Silvano Zanuso, PhD, Centre for Applied Biological & Exercise Sciences, Faculty of Health & Life Sciences, Coventry University, Coventry, UK

**Metabolic Fitness Association, Monterotondo, Rome, Italy**

Paolo Terrana, MD, Ophthalmologist

Adalberto Federici, Podologist

Giovanni Federici, Podologist

Martina Di Nicolò, RN

Gianluca Balducci, Physical Therapist

Enza Spinelli, Exercise Specialist
